# Molecular Characteristics of *Burkholderia pseudomallei* Collected From Humans in Hainan, China

**DOI:** 10.3389/fmicb.2020.00778

**Published:** 2020-05-07

**Authors:** Xiong Zhu, Hai Chen, Sha Li, Li-cheng Wang, Duo-rong Wu, Xu-ming Wang, Ru-shou Chen, Zhen-jun li, Zhi-guo Liu

**Affiliations:** ^1^Sanya People’s Hospital, Sanya, China; ^2^Haikou People’s Hospital, Haikou, China; ^3^Hainan General Hospital, Haikou, China; ^4^The Third People’s Hospital of Hainan Province, Sanya, China; ^5^State Key Laboratory for Infectious Disease Prevention and Control, China Center for Disease Control and Prevention, National Institute for Communicable Disease Control and Prevention, Beijing, China

**Keywords:** melioidosis, *Burkholderia pseudomallei*, molecular characteristics, MLVA, MLST, Hainan

## Abstract

Melioidosis is a common infectious disease in Southeast Asia and Northern Australia. In Hainan, several cases have been reported, but no systematic study has yet been done on the molecular epidemiology profiles of the organism. An investigation of the molecular epidemiology links and population structure of *Burkholderia pseudomallei* would help to better understand the clonally of the isolates and differences among them. In this study, multilocus variable-number tandem repeat analysis (MLVA), and multilocus sequence typing (MLST) were applied to examine the epidemiological relatedness and population structure of 166 *B. pseudomallei* isolates obtained during 2002–2014 in Hainan, China. Both the MLVA_4 and MLST approaches had high discriminatory power for this population, with diversity indices of 0.9899 and 0.9457, respectively. However, the MLVA_4 assay showed a higher discriminatory power than the MLST approach, and a variable-number tandem repeat (VNTR3 933) found by the MLVA approach was the most useful in discriminating strains from this province. A total of 166 strains yielded 99 MLVA_4 genotypes, of which 34 genotypes were shared by 101 isolates, for a clustering rate of 60.8% (101/166), which suggested that some cases may have a common source. Additionally, 65 isolates showed distinct genotypes, indicating that more than 39.2% (65/166) of melioidosis cases in Hainan had epidemiologically unrelated or sporadic characteristics. The 166 isolates were resolved into 48 STs, of which five STs (ST55, -70, -46, -50, and -58) were here found to be predominant. Phylogenetic analysis of 116 isolates conducted using the eBURST v3 segregated the 48 STs into eight groups with ST50 as predicted founder, and 21 STs were found to be singletons, which suggest that the strains in the Hainan region represent a high diversity of ST clones, indicating that many *B. pseudomallei* clone groups are endemic to this region. Moreover, ST50 had 5 SLV, 7 DLV, 6 TLV, and 29 satellite STs and formed a radial expansion pattern, suggesting that the melioidosis epidemic in this study was mainly caused by the clonal expansion of ST 50. Phylogenetic analysis on global scale suggests that China’s isolates are closely related to isolates from Southeast Asia, particularly from Thailand and Malaysia.

## Introduction

Melioidosis, a disease hyperendemic in Northern Australia and Southeast Asia, is caused by the environmental bacterium *Burkholderia pseudomallei* (Wiersinga et al., 2012), and is considered a potential emerging infectious disease in many tropical developing countries (Currie et al., 2008). Melioidosis has a wide variety of symptoms, many of which are shared with other infections, including pyogenic bacterial infection and tuberculosis. Thus, the lack of defining clinical symptoms (Le Tohic et al., 2019) makes melioidosis challenging to diagnose (Shrestha et al., 2019). Most patients are infected through contact with contaminated soil or water. Diabetes, alcoholism, renal insufficiency, and chronic steroid use are important risk factors for the infection by *B. pseudomallei* (Kronsteiner et al., 2019). Melioidosis can have an acute or chronic presentation, and relapse may occur if there is inadequate adherence to treatment or an occult focus of the infection (Currie et al., 2000; Koshy et al., 2019). Based on a geographic information system modeling prediction, the annual incidence of human melioidosis is up to 165,000 cases worldwide, resulting in approximately 89,000 deaths annually (Limmathurotsakul et al., 2016). However, the true incidence of this disease remains difficult to determine. Moreover, due to misdiagnosis and underreporting, it appears likely that there has been a severe underestimation of the incidence of the disease in tropical areas of the world.

Melioidosis has become a significant public health issue in tropical and sub-tropical areas (Kong et al., 2016), including in Hainan province, one of the few tropical areas in China. Although *B. pseudomallei* was detected in the environment in the 1970s, the first human case was not identified until 1989 (Yang, 2000). More than 200 culture-confirmed cases were reported during 2002–2014, and there is evidence that the trend is increasing each year. Hainan is a well-known and important open international island. With a continually developing economy and rising international influence, it is expected to become a major international tourist destination area, a new business center, and an important stage for international exchange. Thus, many individuals are at risk of contracting *B. pseudomallei*, which represents a significant public health concern.

An investigation of the comprehensive molecular epidemiological characteristics of *B. pseudomallei* from clinical data is needed. Multilocus variable-number tandem repeat analysis (MLVA) enables the estimation of epidemiological relatedness among isolated strains, as well as the tracking of pathogens such as *B. pseudomallei* in epidemiological outbreaks at the phylogenetic scale (Pearson et al., 2007; Currie et al., 2009). In addition, multilocus sequence typing (MLST) offers the ability to explore the population structure and evolutionary characteristics. In the present study, a molecular investigation of *B. pseudomallei* strains from clinical samples collected from 2002 to 2014 was performed to estimate the epidemiological relationship and population structure of isolates in Hainan, China.

## Materials and Methods

### Ethics Statement

This study is a retrospective investigation of historical strain collections (2002–2014) using molecular typing methods. The study protocol was approved by the Ethics Committees of the National Institute for Communicable Disease Control and Prevention and the Chinese Center for Disease Control and Prevention. Informed consent was obtained from all patients before testing. Isolated *B. pseudomallei* strains were used to confirm the diagnosis.

### Bacterial Strains and DNA Preparation

Clinical samples from suspected patients were cultured on Columbia blood agar (P0188, Sigma, United States) and incubated at 37°C for 1 week. Colonies of suspected isolates were selected and identified using the Vitek 2 Compact system (Vitek 2 27220, BioMerieux, France), phenotypic features, and 16S rRNA PCR amplification, as previously described (Lowe et al., 2006). A total of 166 strains were characterized in this study, of which 163 were obtained from patients; the remaining three strains were isolated from well water samples. Moreover, there were 14 strains obtained from six infections events (IDs 1–6), five of which (ID 1–5) contained two strains each that were isolated from the same patient, from different clinical samples, or at different point times. ID1 (HNBP040 and HNBP041) and ID4 (HNBP115 HNBP116) each contained two strains that were obtained from different time points. ID2 (HNBP052 and HNBP082), ID3 (HNBP055 and HNBP129), and ID5 (HNBP114 and HNBP135) each contained two strains that were isolated from different clinical samples. However, ID 6 included four strains (HNBP163–HNBP166) from a traceback investigation of one infection event. HNBP163 was obtained from the blood of a patient, and the remaining three (HNBP164–HNBP166) were recovered from well water samples. HNBP164 and HNBP165 were isolated from water from a well located in a patient’s house, and HNBP166 was isolated from the well water of the patient’s neighbor. HNBP134 was recovered from the blood of a patient who was a journalist from Inner Mongolia and conducted news reporting in Sanya City for 2 weeks. DNA was extracted from all strains using a Nucleic Acid Automatic Extraction System (LLXBIO China Ltd., China) with a single loop of fresh bacterial cells according to the manufacturer’s instructions. DNA concentrations were measured by UV spectrophotometry (NanoDrop 2000, Thermo Fisher, United States). The DNA extracted from all isolates was stored at −20°C.

### MLVA Approach

The MLVA_4 genotyping assay was performed as previously described (Currie et al., 2009). Briefly, the four higher-variation variable-number tandem repeat (VNTR) loci 2341, 389, 1788, and 933 were chosen for MLVA genotyping (U’Ren et al., 2007). A 20-μL amplification system was applied, and all PCR involved an initial denaturation at 95°C for 5 min, followed by 30 cycles at 94°C for 30 s, 68°C for 30 s, and 72°C for 30 s, with a final extension step at 72°C for 5 min. Next, 5 μL of each PCR product was separated by gel electrophoresis, and the bands of the expected amplicons from the four loci were denatured and resolved by capillary electrophoresis using an ABI Prism 3130 automated fluorescent capillary DNA sequencer (Applied Biosystems, United States). The fragments were sized following comparison with a ROX (carboxy-X-rhodamine)-labeled molecular ladder (MapMaker 1000; BioVentures Inc., Murfreesboro, TN, United States) and Gene Mapper software version 4.0 (Applied Biosystems). The fragment sizes were subsequently converted to repeat unit numbers using a published allele numbering system. The MLVA_4 data were imported into BioNumerics 7.6 software (Applied Maths, Sint-Martens-Latem, Belgium) for cluster analysis (Supplementary Table S1). The molecular epidemiological relatedness of isolates was evaluated using a matrix of the pairwise differences for the 4 VNTR loci, with a dendrogram produced using the unweighted pair group method with arithmetic averages (UPGMA).

### MLST Assay

Multilocus sequence typing assays were performed as previously described (Price et al., 2016a). Each allele was assigned a different number, and the allelic profile (a string of seven integers) was used to define the sequence type (ST) for that isolate (Supplementary Table S1). The allelic profiles of the isolates were imported into BioNumerics version 7.6, and the relatedness of the isolates was displayed as a dendrogram using the matrix of pairwise differences in the allelic profiles and UPGMA clustering. The genetic diversity and discriminatory power of each typing method were calculated based on the Hunter-Gaston diversity index (HGDI), according to a previously published method (Hunter, 1990).

The similarity of MLST profiles of isolates identified in this study or elsewhere in China (Supplementary Table S2) in *B. pseudomallei* MLST database was assessed using eBURST software as described previously (Kamthan et al., 2018) STs (Supplementary Table S3). The relationship of China STs to the global collection of STs was assessed using the eBURST algorithm with PHYLOViZ 2.0 (Nascimento et al., 2017) available at MLST site^1^. All MLST profiles have been submitted to the MLST DB^2^.

## Results

### Demographic and Clinical Characteristics of Patients

A total of 166 strains were investigated in this study; three strains were from environmental samples (well water), and 163 *B. pseudomallei* isolates were obtained from clinical samples (158 patients). Ten of the strains were obtained from five patients, each harboring two strains. Regarding the 158 *B. pseudomallei* isolates obtained from 158 individual patients, 59% (94/158) of the patients were engaged in livestock farming and related work. The mean age of these 158 patients was 48.6 years (range: 1–82 years), and the ratio of males (*n* = 132) to females (*n* = 26) was 5.1 (Supplementary Table S4). The patients had wide-ranging symptoms: 61 (38.6%) had sepsis, 58 (36.7%) had pulmonary infections, and 34 (21.5%) had local abscesses. Moreover, 73 (46.2%) patients had known diabetes risk factors. A total of 117 (75.3%) patients were cured, 39 (24.7%) died, and the other two had unknown outcomes. Among the 39 patients who died, 18 (46.2%) had diabetes, 27 (69.2%) had septicemia, and 19 (48.7%) had pulmonary infections. The age range of the patients who died was 1–77 years, and the predominant age range was 40–50 years.

### Bacterial Identification and Profile Distribution of Strains

**FIGURE 1:**
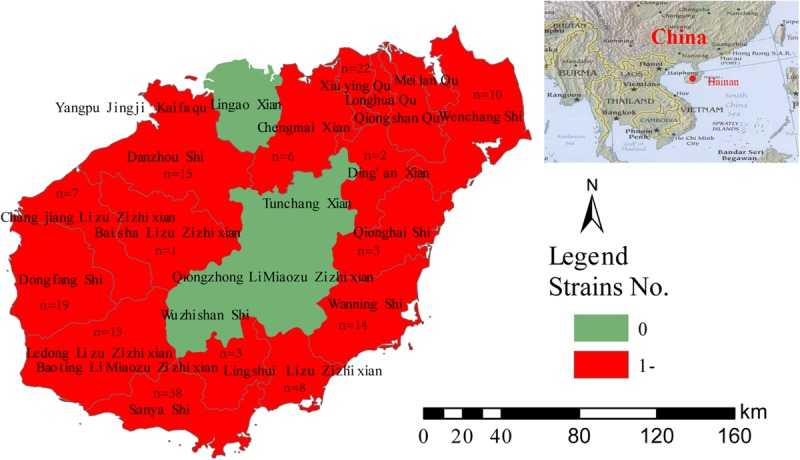
Geographic distribution of 161 clinical *B. pseudomallei* strains.

A total of 163 strains were collected from clinical patient samples, including blood, secretions, and pus, and three strains were isolated from well water samples. All the strains were identified as *B. pseudomallei*, which is a gram-negative, bipolar, aerobic, motile, and rod-shaped bacterium. Colonies of the strains were wrinkled, had a robust metallic appearance, and emitted an acrid, earthy smell. Of the 166 strains collected from 2002 to 2014, 65.7% (109/166) were obtained between 2010 and 2012 (Supplementary Table S5). Five strains were isolated from the blood of patients from Fujian (*n* = 2), Inner Mongolia (*n* = 1), Hunan (*n* = 1), and Russia (*n* = 1); these patients lived in Hainan for a period before they became sick. Other strains were obtained from 14 counties in Hainan Province (Figure 1).

### VNTR and Allele Diversity Profiles

In this study, the results of *B. pseudomallei* isolate diversity analysis confirmed that both the MLVA_4 and MLST approaches offer high discriminatory power for this population, with diversity indices of 0.9899 and 0.9457, respectively, although the former displayed higher discriminatory power than the latter (Table 1). We sorted 166 strains into 48 STs in the MLST assay and 99 different MLVA genotypes in the MLVA_4 assay. The HGDI for MLVA was the highest (0.9025) for VNTR3 (933), which had 15 allele types. The allele numbers for VNTR1 (2341), VNTR2 (1788), and VNTR4 (389) by MLVA were 11, 9, and 14, respectively, and the polymorphism levels of all loci were ≥0.8207. In MLST, the highest number of alleles was found for the *gmhD* locus (10), which also had the highest HGDI (0.7925), followed by *narK* with eight alleles, *gltB*, and *lepA* with five alleles, and the remaining loci with three alleles (Table 1).

**TABLE 1:** Allelic types and Hunter-Gaston diversity index (HGDI) of *B. pseudomallei* for 11 typing loci in this study.

**Approach**	**Loci**	**Distribution profile of allelic types (numbers of strains)**	**HGDI values**
MLVA	VNTR4 (389)	2 (26), 3 (39), 4 (55), 5 (3), 6 (12), 7 (11), 8 (5), 9 (11), 10 (3), 11 (1), 12 (5), 13 (1), 14 (1), 21 (2)	0.8207
	VNTR2 (1788)	3 (24), 4 (33), 5 (42), 6 (36), 7 (4), 8 (12), 9 (3), 10 (11), 12 (1)	0.8229
	VNTR1 (2341)	3 (27), 4 (2), 5 (6), 6 (32),7 (15), 8 (44), 9 (5),10 (17),11 (10), 12 (6), 13 (2)	0.8451
	VNTR3 (933)	4 (7), 5 (3), 6 (1), 7 (6), 8 (8), 9 (14), 10 (22), 11 (29), 12 (26), 13 (13), 14 (11), 15 (9), 16 (8), 17 (3), 18 (6)	0.9025
	MLVA_4	–	0.9899
MLST	lipA	1 (137), 5 (25), 8 (4)	0.2974
	ace	1 (58), 3 (107), 4 (1)	0.4652
	gltB	1 (116), 2 (24), 3 (1), 4 (37), 12 (12)	0.5506
	ndh	1 (77), 3 (77), 6 (12)	0.5679
	lepA	1 (98), 2 (17), 3 (45), 4 (5), 68 (1)	0.5700
	narK	1 (6), 2 (14), 3 (29), 4 (99), 6 (2), 9 (6), 22 (6), 29 (4)	0.6057
	gmhD	2 (60), 3 (36), 4 (9), 5 (19), 6 (4), 11 (17), 13 (6), 14 (1), 28 (13), 36 (1)	0.7925
	MLST	–	0.9457

*HGDI, Hunter-Gaston diversity index (); MLVA, Multiple-locus variable-number tandem repeat analysis; MLST, Multilocus sequence typing; and VNTR, Variable-number tandem repeat analysis.*

### Epidemiological Characteristics Based on MLVA Genotyping

**FIGURE 2:**
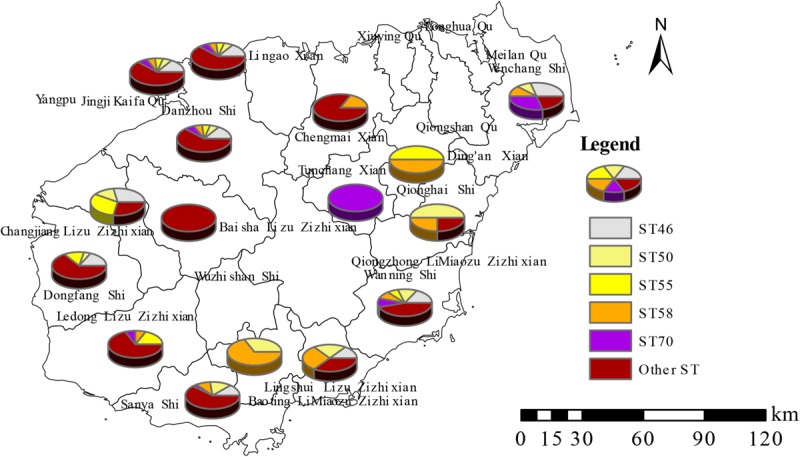
Distribution characteristics of five predominant STs in Hainan Province.

**FIGURE 3:**
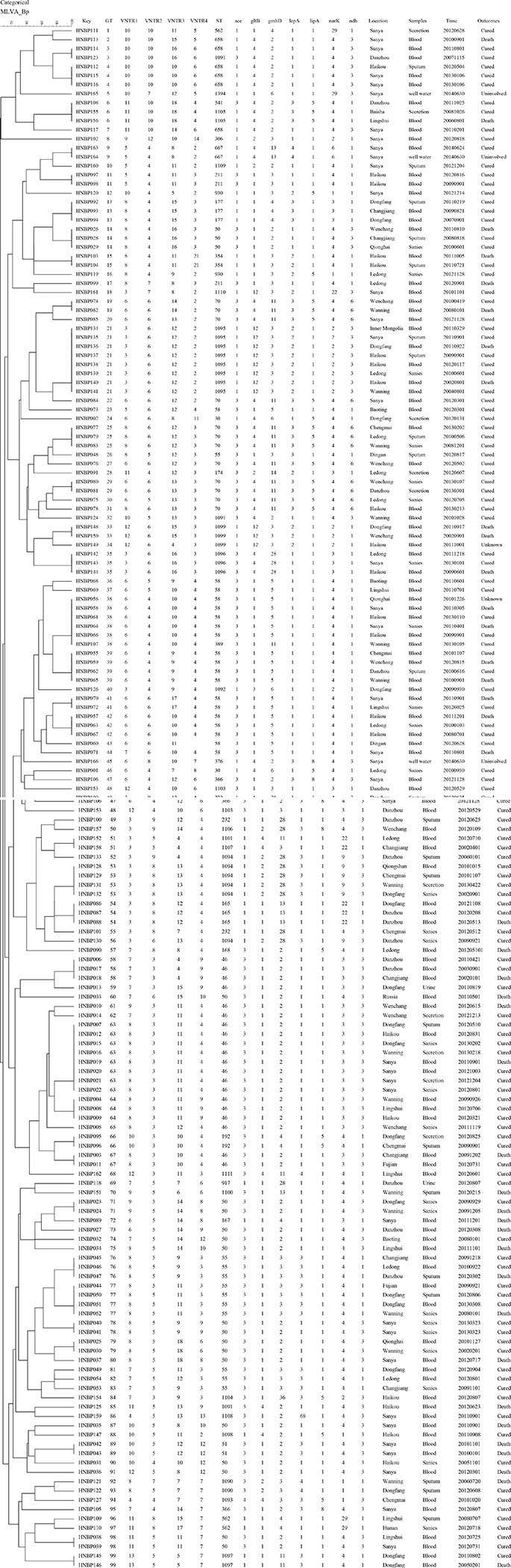
A dendrogram of the 166 *B. pseudomallei* strains showing the strain identification features, MLVA_4 and MLST characters’ values, their geographical origins, and the year of isolation.

A total of 166 strains were sorted into 99 different MLVA_4 genotypes; 34 genotypes were found to be shared by 101 strains, accounting for 61% (Figures 2, 3). Seven shared MLVA_4 genotypes (GT11, 38, 42, 54, 63, 77, and 89), and each one of them was comprised of two strains from identical locations and similar times (Table 3). Five shared MLVA_4 genotypes (GT 21, 38, 39, 53, and 63), including strains with identical STs and from two to five different regions over a long period (Table 4). Notably, MLVA_4 genotype 21 is shared by eight strains obtained from five areas and isolated 10 years apart. The other 65 strains harbor unique genotypes, with each genotype representing a single strain. Moreover, the MLVA and MLST data for six individual infection events (IDs 1–6) showed that the strains with IDs 1 and 4 have identical MLVA_4 genotypes and the same ST; the strains with IDs 2, 3, and 5 exhibit different MLVA_4 profiles and STs (Table 2). A total of four strains were isolated from the ID 6 infection event on a farm in Saya. The HNBP163 strain, derived from the blood of one patient, has an MLVA_4 genotype (GT9) and ST (ST667) identical to those of HNBP164, which was isolated from the well water of the same patient. However, there was a significant difference between the strains (HNBP165 and HNBP166) collected from the two other well water samples (Table 2). HNBP134 has an MLVA_4 genotype and ST identical to those of strains from five different regions in Hainan Province. Two strains from Fujian Province exhibit different MLVA_4 genotypes and STs. GT60 and GT97 each represent unique strains obtained from the blood of patients from Russia and Hunan, respectively.

**TABLE 2:** Information on scene epidemiology, MLVA and MLST for six infection events (ID1–6).

**ID**	**Key**	**GT (MLVA_4)**	**ST**	**VNTR1**	**VNTR2**	**VNTR3**	**VNTR4**	**Ace**	**GltB**	**GmhD**	**LepA**	**LipA**	**NarK**	**Ndh**	**Location**	**Sources**	**Time**	**Outcomes**
1	HNBP040	GT78	50	8	5	9	9	3	1	2	1	1	4	3	Sanya	Pus	3/23/2013	Cured
	HNBP041			8	5	9	9	3	1	2	1	1	4	3				
2	HNBP052	GT77	55	8	5	11	3	3	1	3	3	1	4	1	Wanning	Pus	1/1/2008	Death
	HNBP082	GT19	70	6	6	14	2	3	4	11	3	5	4	6		Blood		
3	HNBP055	GT39	58	6	4	9	4	3	1	5	1	1	4	1	Chengmai	Blood	11/7/2010	Cured
	HNBP129	GT53	1094	3	8	13	4	1	2	28	3	1	9	3		Sputum		
4	HNBP115	GT4	658	10	10	10	6	1	4	2	1	1	4	3	Sanya	Blood	1/6/2013	Cured
	HNBP116			10	10	10	6	1	4	2	1	1	4	3				
5	HNBP114	GT3	658	10	10	16	6	1	4	2	1	1	4	3	Sanya	Blood	8/1/2011	Cured
	HNBP135	GT21	1095	3	6	12	2	1	12	3	2	1	2	3		Sputum	9/1/2011	
6	HNBP163	GT9	667	5	4	8	2	1	4	13	4	1	6	1	Sanya	Blood	6/24/2014	Cured
	HNBP164			5	4	8	2	1	4	13	4	1	6	1		Well water	6/30/2014	Uninvolved
	HNBP165	GT5	1394	10	7	12	5	1	1	6	1	1	29	3		Well water		
	HNBP166	GT45	376	6	8	10	7	1	4	2	3	8	4	3		Well water		

**TABLE 3:** Strains with shared genotypes and the same source of infection.

**Key**	**GT**	**Host**	**Location**	**Time**	**Source**	**Location**
HNBP097	11	patient	Haikou	20120816	The same rice paddy for work	Identical towns
HNBP098			Haikou	20090901		
HNBP058	38		Sanya	20110305	The orchard beside a ravine stream for work	The same village
HNBP064			Sanya	20110401		
HNBP057	42		Haikou	20111201	The same rice paddy for work	identical towns
HNBP067			Haikou	20080701		
HNBP087	54		Danzhou	20120208	The same rice paddy for work	identical towns
HNBP088			Danzhou	20120513		
HNBP020	63		Sanya	20121003	The same rice paddy for work	identical towns
HNBP021			Sanya	20121204		
HNBP050	77		Dongfang	20120806	The orchard beside a ravine stream for work	The same village
HNBP051			Dongfang	20130308		
HNBP042	89		Sanya	20101101	The same rice paddy for work	identical towns
HNBP043			Sanya	20100101		

**TABLE 4:** Strains with an identical MLVA_4 genotype and ST from distinct regions.

**Key**	**GT**	**ST**	**Location**	**Time span**
HNBP135	21	1095	Sanya	2002–2012
HNBP136		1095	Dongfang	
HNBP137		1095	Haikou	
HNBP138		1095	Haikou	
HNBP139		1095	Ledong	
HNBP140		1095	Haikou	
HNBP141		1095	Wanning	
HNBP056	38	58	Qionghai	2009–2013
HNBP058		58	Sanya	
HNBP061		58	Haikou	
HNBP064		58	Sanya	
HNBP066		58	Haikou	
HNBP055	39	58	Chengmai	2010–2012
HNBP059		58	Wenchang	
HNBP062		58	Danzhou	
HNBP065		58	Wanning	
HNBP128	53	1094	Qiongshan	2002–2013
HNBP129		1094	Chengmai	
HNBP131		1094	Wanning	
HNBP132		1094	Dongfang	
HNBP007	63	46	Dongfang	2011–2013
HNBP012		46	Haikou	
HNBP015		46	Dongfang	
HNBP016		46	Wanning	
HNBP019		46	Sanya	
HNBP020		46	Sanya	
HNBP021		46	Sanya	
HNBP022		46	Sanya	

### MLST Analysis and Regions Distribution Profiles of ST

**FIGURE 4:**
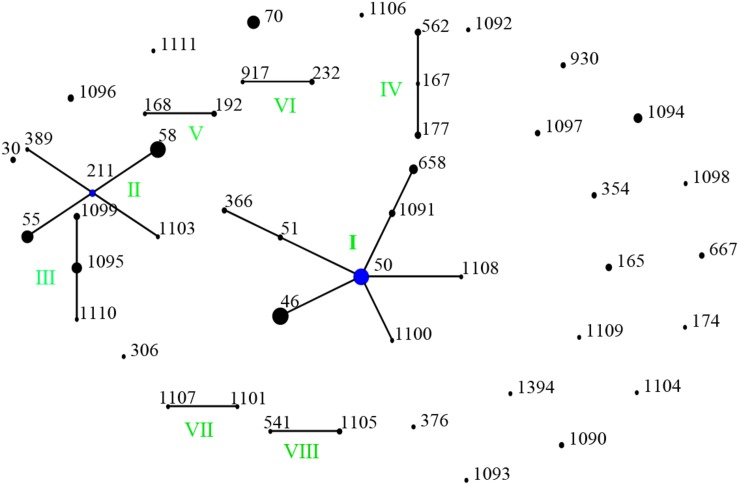
Genetic relationship of 166 *B. pseudomallei* isolates of this study using eBURST. (Each black dot represents single genotype, and blue dot refers to group founder). Strains from this study formed eight (I-VII) ST groups.

All 166 strains were resolved into 48 STs, three of which (ST167, −168, and −389) were singleton STs that had not been previously reported from Hainan. The other STs are identical to those previously reported. Nineteen STs were observed in Sanya, 13 in Dongfang and Haikou, 12 in Danzhou, 11 in Ledong and Wanning, and six in Lingshui and Wenchang. Other regions had one to five different STs (Supplementary Table S6). Five STs (ST55, −70, −46, −50, and −58) were predominant in the population in this study (Figure 4). The dominant STs, which occurred in ≥11 cases, were ST46 (twenty cases; 12.0%), ST50 and ST58 (nineteen; 11.4%), ST70 (twelve; 7.2%), and ST55 (eleven; 6.6%). These five STs accounted for 48.8% of all cases, and the remainder were associated with one to eight cases. Two STs, ST168, and -389, are single-locus variations of ST48. ST167 is a single-locus variation of ST562. Moreover, twelve different STs, including four cases of ST50 and two cases of both ST55 and −58, were responsible for 18 deaths. Eight STs (ST46, −50, −55, −58, −70, −658, −1094, and −1095) each included more than five strains, and these STs were found to have one to thirteen similar or identical MLVA_4 genotypes (Supplementary Table S7).

### Genetic Relatedness Among STs From China and Global Collection

**FIGURE 5:**
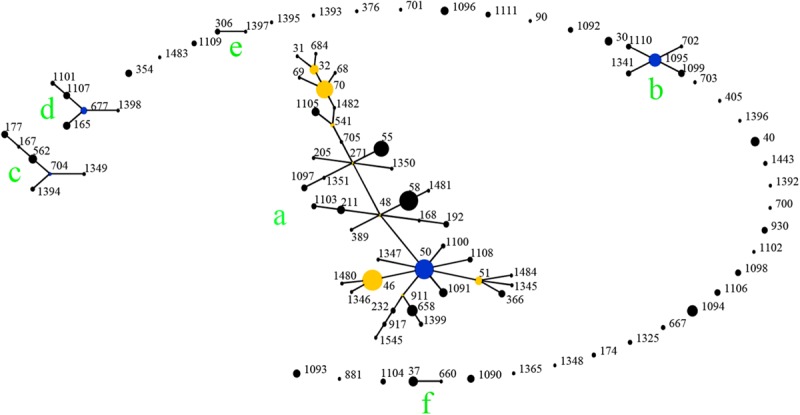
Genetic relationship of all China *B. pseudomallei* isolates (*n* = 479) using eBURST. (Blue dot refers to group founder, and yellow dot refers to sub-group founder. Each black dot represents single genotype. Re-sampling for bootstrapping = 10, 000; minimum number of identical loci for group definition = 6; minimum number of SLV for subgroup definition = 3). 479 isolates from China were clustered into 95 STs, and the 62 STs were clustered in six groups **(a–f)**.

**FIGURE 6:**
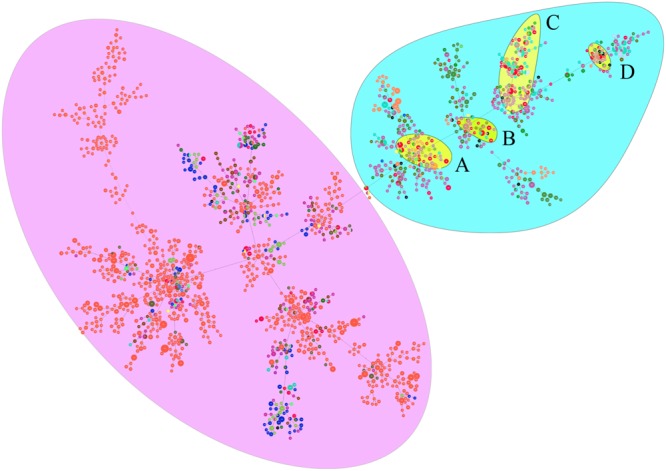
PHYLOViZ analysis showing the genetic relationship among global collection of sequence types (STs) of 6161 *B. pseudomallei*. Each dot represents a distinct ST. Oceania and Southeast Asian dominant STs are shaded in purple and light blue, respectively. China STs (shaded in yellow) cluster in four groups—All four groups cluster with STs from Southeast Asia. STs from China are shown in red. Different colored dots represent STs from Australia (orange), Thailand (light violet), Malaysia (sky blue), other countries (deep olive green), India (blue), Cambodia (brown), Vietnam (mint green), Sri Lanka (grass green), Singapore (gray), Bangladesh (yellow), Burma (light green), Laos (black), Turkey (indigo), Philippines (purple), and Japan (chartreuse). The majority of Chinese isolates grouped into four groups **(A–D)**.

When analyzed using eBURST, the 48 STs evaluated in this study formed eight groups (I–VIII) with ST50 as predicted founder, and 21 STs were found to be singletons (Figure 4). These two groups (I and II) form a radial expansion pattern. ST50 had 5 SLV, 7 DLV, 6 TLV, and 29 satellite STs. Based on the eBURST analysis, 479 isolates from China were clustered into 95 STs, and the 62 STs were clustered in six groups (a–f) with the remaining 33 STs being singletons. ST50, the predicted founder ST in this population (Figure 5), had a frequency of 42 with 8 SLV, 13 DLV, 15 TLV, and 58 satellite STs. ST46 and ST51 were subgroup founders. The present study expanded the clonal cluster of China isolates by adding more branching STs. When Chinese STs were analyzed using PHYLOViZ against the global collection of 6046 isolates in the *B. pseudomallei* MLST isolate database (as on 20th May 2020), the majority of Chinese isolates grouped into four groups (A–D; Figure 6) and were almost exclusively clustered in the Southeast Asia clade. It appears that there are a few outliers (e.g., ST37, −660, −1099, −1101, and −1107) that are distantly related to the majority of Hainan STs. China’s isolates appeared to be different from the Oceania cluster (Australia) and grouped closer to isolates from Southeast Asia, e.g., Thailand, Malaysia, and Vietnam (Figure 6).

## Discussion

In this study, pneumonia and sepsis were the most common clinical symptoms of melioidosis patients; 46.2% of patients had known diabetes risk factors, and those with diabetes mellitus have a more than 10-fold higher susceptibility to melioidosis (Currie et al., 2004; Kasantikul et al., 2016). A high death rate (25.0%) was observed, and 69.2% (27/39) of the deceased patients were septic. Septic shock cases have a greater than 90% mortality rate (Cheng and Currie, 2005; Kingsley et al., 2016). Thus, early diagnosis and timely treatment are crucial to obtaining satisfactory therapeutic effects. A total of 65.7% (109/166) of the strains in this study were obtained between 2010 and 2012. These data suggest that this disease exhibited an increasing trend after 2010, in part due to greater awareness and improved diagnostics.

Moreover, these strains were found in 14 areas (of a total of 22 administrative areas) of Hainan Province, indicating a wide distribution for these organisms, although the majority of the strains were detected in coastal areas, where the population density is higher than in center counties (cities). Moreover, due to poor economic resources, many patients from central regions had few chances to be treated. The highest isolation rate from soil samples was observed in the southern coastal region (Dong et al., 2018). The positivity rate for *B. pseudomallei* in coastal areas or wet rice fields is higher than in mountainous regions in many parts of the globe, rendering the former areas serious epidemic areas for melioidosis (Hsun-Pi et al., 2007; Archana et al., 2014).

MLVA_4 and MLST have higher discrimination power than do ribotyping and RAPD based on PCR amplification (Currie et al., 2009). The MLVA_4 assay showed a higher discriminatory power than MLST (Pearson et al., 2007). The HGDI for MLVA was the highest (0.9025) for VNTR3, with fifteen allele types, suggesting that VNTR3 in the MLVA approach is most useful for discriminating among strains from this province. Furthermore, the most variable locus in MLST was *gmhD*, with ten alleles. This locus may play a dominant role in the population diversity of *B. pseudomallei* in this region (Wang et al., 2016). Moreover, *B. pseudomallei* is a highly recombinogenic species, and recombination events are a key factor for genetic differentiation (Price et al., 2015). The strains from many STs corresponded to three to thirteen similar or related MLVA_4 genotypes, suggesting that MLVA_4 can be used to discriminate closely related clones of *B. pseudomallei*.

More than 60.0% (101/166) of the strains were in clusters, suggesting that some cases may have a common source. Currie et al. (2009) found that MLVA_4 was able to distinguish epidemiologically unlinked strains that were identical by MLST and PFGE, although the isolates from confirmed point-source outbreaks were either identical or clustered closely. Patients carrying *B. pseudomallei* with seven shared MLVA_4 genotypes (GT11, −38, −42, −54, −63, −77, and −89) represent identical sources of infection, of which five patients (GT11, −42, −54, −63, and −89) shared the same rice paddy for work. The patients carrying the remaining two genotypes (GT38 and -77) shared an orchard beside a ravine stream, suggesting that these strains are epidemiologically related. In addition, five shared MLVA_4 genotypes (GT21, −38, −39, −53, and −63) included strains with identical STs from different regions over a long time span; these data show an ongoing spread of melioidosis not only within a specific region but also among different regions of Hainan. Nonetheless, without WGS, it is difficult to define how closely related these identical genotypes are without shared epidemiology. Additionally, 65 isolates showed distinct genotypes, indicating that more than 39.2% (65/166) of the melioidosis cases in Hainan had epidemiologically unrelated sporadic characteristics. Two strains from the same patient (IDs 1 and 4) have identical MLVA_4 genotypes and STs, suggesting the occurrence of a single *B. pseudomallei* infection. Different MLVA_4 profiles and STs were observed in pairs of strains from single infection events (IDs 2, 3, and 5), suggesting that these cases may have been infected by strains from two different *B. pseudomallei* colony populations or that variation occurred within the strains (Pearson et al., 2007).

Moreover, previous research confirmed that a single unchlorinated water source harboring multiple *B. pseudomallei* strains was linked to an outbreak (Sarovich et al., 2017). In this study, HNBP163, which was isolated from a patient, has an MLVA_4 genotype (GT9) and ST (667) identical to that of a strain (HNBP164) from a water well located in the patient’s house, suggesting that the source of infection, in this case, was the well water. In addition, strains with two different STs (ST1394 and ST376) were isolated from the same well water sample, and five loci differences were found between ST1394 and ST376. These data provide strong evidence that two strains with distinct STs can be isolated from the same well. Moreover, strains from patients from Inner Mongolia, Fujian, and Hunan had an MLVA_4 genotype and ST identical to those of strains from Hainan. These patients presented to the hospital after traveling to Hainan. Combined field epidemiology suggests that these patients may have had travel-associated infections. HNBP033 (GT60) was obtained from Russia, presenting unique MLVA_4 genotypes, and a further survey of these isolates by WGS may help better trace the sources of infection (Currie et al., 2015; McRobb et al., 2015).

A total of 166 strains were divided into 48 STs, 5 STs accounted for 48.8% of all cases, suggesting that the most common STs are overrepresented in the isolate population associated with disease (Vesaratchavest et al., 2006). When the STs were analyzed using eBURST, the 48 STs were divided into 8 groups and 21 singletons, suggesting that the strains in the Hainan region represent a high diversity of ST clones (Wang et al., 2016). ST50, the predicted founder ST in this study, connected to two dominant ST58 and ST46 and most of the STs by SLV, DLV, or TLV. ST50 is also the predicted founder ST in the Chinese population, and it had a frequency of 42 with 8 SLV, 13 DLV, 15 TLV, and 58 satellite STs. These data suggest that the melioidosis epidemic in China was mainly due to the clonal expansion of ST 50. ST 50 is common in Malaysia and Thailand. Malaysian STs were clustered into a single group with ST50 as the predicted founder (Zueter et al., 2018). ST167 is a single-locus variation of the ST562 type. It was likely imported into Australia from somewhere in Asia (Price et al., 2016b). The other two newly detected STs, 168 and −389, are single or (double)-locus variations of ST48, which is of Thai origin (McCombie et al., 2006). These regions are geographically close, suggesting a potential molecular epidemiology connection between strains from the ST50 clone complex in these regions. The majority of China isolates clustered in Southeast Asia clade suggest the possible dissemination of melioidosis across these Asian countries. There appear to be a few outliers that are distantly related to the majority of Hainan STs and group in the Oceania lineage. There are also three predominant STs (ST46, −58, and −70). These included both strains from this study and strains from Australia, suggesting the probable travel of these predominant STs over time in a global context. The communication and commerce activities between countries may promote the spread of *B. pseudomallei* strains with different genetic backgrounds. Exploring the geographical expansion and spread of STs among countries and regions is essential to better understand the epidemiology of melioidosis at the global level (Cheng et al., 2008). Another possible explanation is the possibility of ST homoplasy among strains from distinct regions, whereby isolates have the same ST, but do not have shared ancestry and may be distantly related at the whole-genome level. Thus, future work includes performing whole-genome sequencing on all isolates, which is much higher resolution compared to MLST and MLVA (Gee et al., 2017).

Our study has some limitations. First, the data used were collected from passive diagnoses that might have been influenced by case definitions, laboratory tests, or each physician’s understanding of the disease. Second, due to variability in the number of strains collected among the different counties and years, further research with additional strains is essential. Third, a limited number of environmental samples were included (*n* = 3), and thus extensive environmental sampling is needed to accurately determine the distribution of the STs. This will be vital for source attribution, in order to determine where patients are gaining infection, as well as for guiding public health initiatives and remediation activities on patient property in other regions around Hainan.

## Conclusion

In conclusion, a molecular investigation of *B. pseudomallei* during 2002–2014 was performed in this study. An MLVA_4 assay confirmed that a significant proportion of melioidosis in this province was due to multiple contaminations from a limited number of sources. Moreover, the melioidosis cases in Hainan showed epidemiologically unrelated or sporadic characteristics. Our results demonstrate high diversity was observed in the Hainan strain population, and extensive ST sharing between the strains from this study and those from Thailand, Malaysia, and Vietnam. Determining the homoplasy between the strains of the same ST and MLVA_4 genotype in different geographical locations using WGS is essential to better understand the epidemiology of melioidosis at the global level.

## Data Availability Statement

The raw data supporting the conclusions of this article will be made available by the authors, without undue reservation, to any qualified researcher.

## Ethics Statement

This study is a retrospective investigation of historical strain collections using modern typing methods. The study protocol was approved by the Ethics Committees of the National Institute for Communicable Disease Control and Prevention and the Chinese Center for Disease Control and Prevention. Informed consent was obtained from all the patients prior to testing. *B. pseudomallei* strains isolated were using for confirmation diagnosis.

## Author Contributions

XZ performed most of the strain isolation and MLVA typing. ZGL performed the MLVA cluster analysis and MLST typing and drafted the manuscript. HC, SL, and LCW performed the strain biotyping. DRW and XMW prepared the DNA samples. RSC and ZJL participated in the design of the study and critically reviewed the manuscript. ZGL and ZJL participated in the design of the study and managed the project. All authors read and approved the final manuscript.

## Conflict of Interest

The authors declare that the research was conducted in the absence of any commercial or financial relationships that could be construed as a potential conflict of interest.

## Footnotes

1 http://pubmlst.org/bpseudomallei

2 https://pubmlst.org/bpseudomallei/

## Supplementary Material

TABLE S1 Strain identification codes (Key), GT (MLVA_4), MLST characteristics, location, sources, year of isolation, and outcomes for 166 *B. pseudomallei* isolates.

TABLE S2 The 479 Chinese *B. pseudomallei* strains used to eBURST analysis.

TABLE S3 The 6161 *B. pseudomallei* strains used to phylogenetic analysis on a global scale.

TABLE S4 Patient demographic features.

TABLE S5 Distribution of 166 *B. pseudomallei* strains from 2002 to 2014.

TABLE S6 Distribution characteristics of 48 STs of *B. pseudomallei* in Hainan Province.

TABLE S7 Links between MLST and MLVA_4 genotyping approaches.

## Abbreviations

DLV: Double-locus variants
MLST: Multilocus sequence typing
MLVA: Multiple-locus variable-number tandem repeat analysis
MST: Minimum spanning tree
PCR: Polymerase chain reaction
RAPD: Random amplified polymorphic DNA
SLV: Single-locus variants
ST: Sequence type
VNTR: Variable-number tandem repeat analysis
WGS: Whole-genome sequencing
TLV: Triple-locus variants.
